# Supplementary material for: Deficiency of gluconeogenic enzyme PCK1 promotes metabolic-associated fatty liver disease through PI3K/AKT/PDGF axis activation in male mice
Source: Nat Commun. 2023 Mar 14;14:1402. doi: 10.1038/s41467-023-37142-3 (PMC10015095; doi:10.1038/s41467-023-37142-3)
Supplement: Supplementary file 1 — Supplementary Information [file 41467_2023_37142_MOESM1_ESM.pdf]

Deficiency of gluconeogenic enzyme PCK1 promotes metabolic-associated fatty liver disease (MAFLD) through PI3K/AKT/PDGF axis activation in male mice

Qian Ye[1,#] Yi Liu[1,#], Guiji Zhang[1,#], Haijun Deng[1,#], Xiaojun Wang[2,#], Lin Tuo[3,#], Chang Chen[4], Xuanming Pan[1], Kang Wu[1], Jiangao Fan[5], Qin Pan[5], Kai Wang[1,*], Ailong Huang[1,*], Ni Tang[1,*]

[1]Key Laboratory of Molecular Biology for Infectious Diseases (Ministry of Education), Institute for Viral Hepatitis, Department of Infectious Diseases, The Second Affiliated Hospital, Chongqing Medical University, Chongqing, China

[2]Institute of Hepatobiliary Surgery, Southwest Hospital, Third Military Medical University (Army Medical University), Chongqing, China

[3]Department of Infectious Disease, Hospital of the University of Electronic Science and Technology of China and Sichuan Provincial People's Hospital, Chengdu, China

[4]Institute of Life Sciences, Chongqing Medical University, Chongqing, China

[5]Department of Gastroenterology, Xin Hua Hospital, School of Medicine, Shanghai Jiao Tong University, Shanghai, China

[#] These authors contributed equally：Qian Ye, Yi Liu, Guiji Zhang, Haijun Deng, Xiaojun Wang, Lin Tuo

***Corresponding author:** Ni Tang, Ailong Huang, Kai Wang, Key Laboratory of Molecular Biology for Infectious Diseases (Ministry of Education), Institute for Viral Hepatitis, Department of Infectious Diseases, The Second Affiliated Hospital, Chongqing Medical University, Chongqing, 400016, China. Tel: 86-23-68486780, Fax: 86-23-68486780, E-mail: nitang@cqmu.edu.cn (N.T.), ahuang@cqmu.edu.cn (A.H.), wangkai@cqmu.edu.cn (K.W.)

Supplementary Figures and Figure Legends

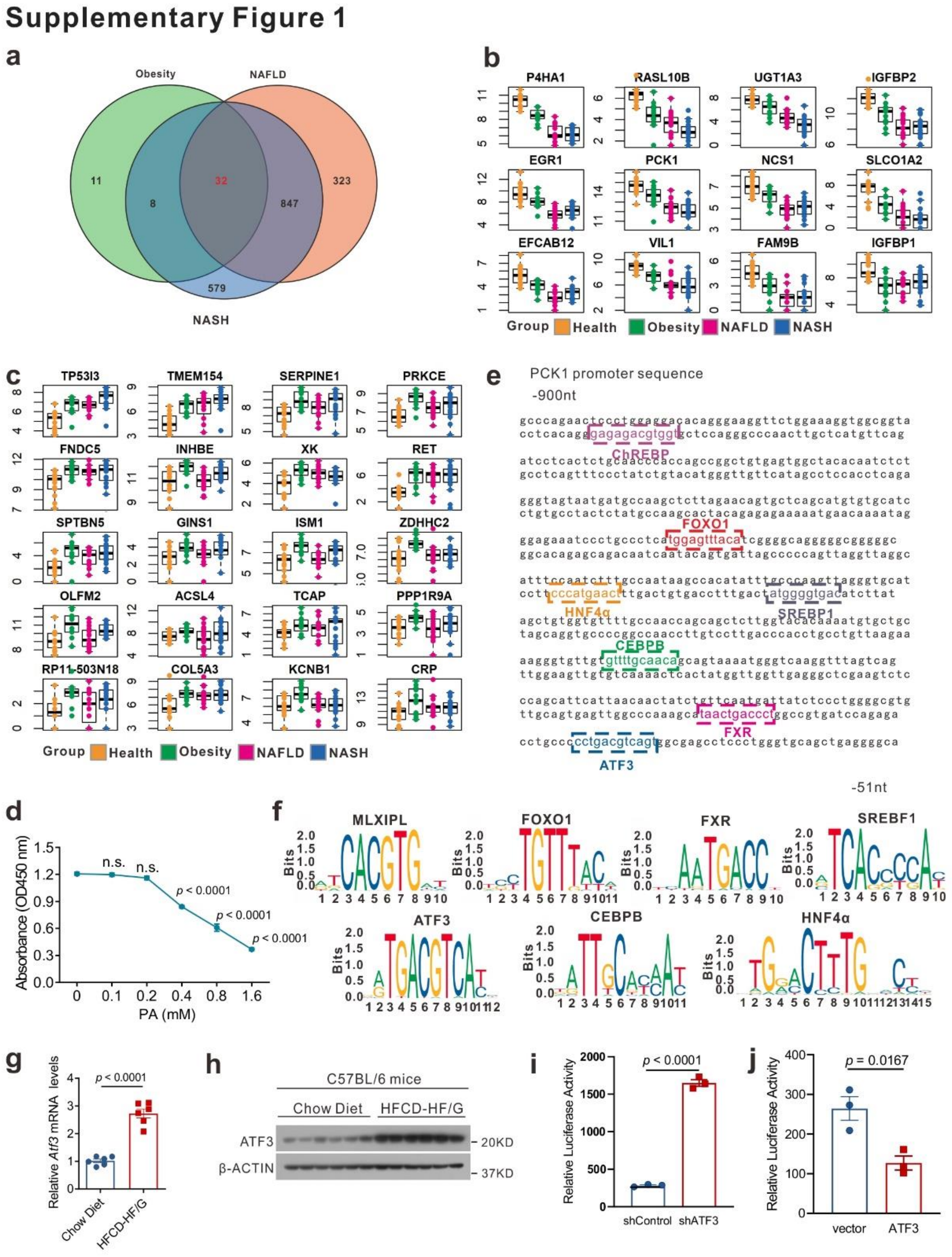

Supplementary Fig. 1 PCK1 is downregulated in NASH patients due to ATF3 upregulation in HFCD-HF/G induced mouse model. a Venn Diagram showing a total of 32 genes were significantly changed in patients with obesity,

NAFLD, and NASH. **b** 12 genes were significantly downregulated in health (n = 14), obesity (n = 12), NAFLD (n = 15), and NASH (n = 16). **c** 20 genes were significantly upregulated in health (n = 14), obesity (n = 12), NAFLD (n = 15), and NASH (n = 16). The box plots show the medians (middle line) and the first and third quartiles (boxes), whereas the whiskers show 1.5× the IQR above and below the box. Unpaired, two-sided Mann–Whitney U test *P* values are depicted in the plots, and the significant *P* value cutoff was set at 0.05. **d** Cell proliferation was assessed by a CCK8 assay (n = 3). **e** Illustration of the predicted transcription factor binding sites in the 0.9 kb *PCK1* promoter region using JASPAR. **f** Potentially regulatory binding sequences of the transcription factors generated by JASPAR. **g, h** The mRNA (**g**) and protein levels (**h**) of ATF3 in mice fed with HFCD-HF/G (n = 6). **i, j** Luciferase assay of *PCK1* promoter constructs in MIHA cells knockdown (**i**) or overexpression (**j**) of ATF3 (n = 3). Results were obtained as relative luciferase activity against the activity of pGL3-Basic. Each figure represents at least three independent experiments. n was the number of biologically independent mice. Data expressed as mean ± SD; n.s., not significant. *P* values obtained via 2-tailed unpaired Student's t tests or one-way ANOVA with Tukey's post hoc test. Source data are provided as a Source Data file.

Supplementary Figure 2

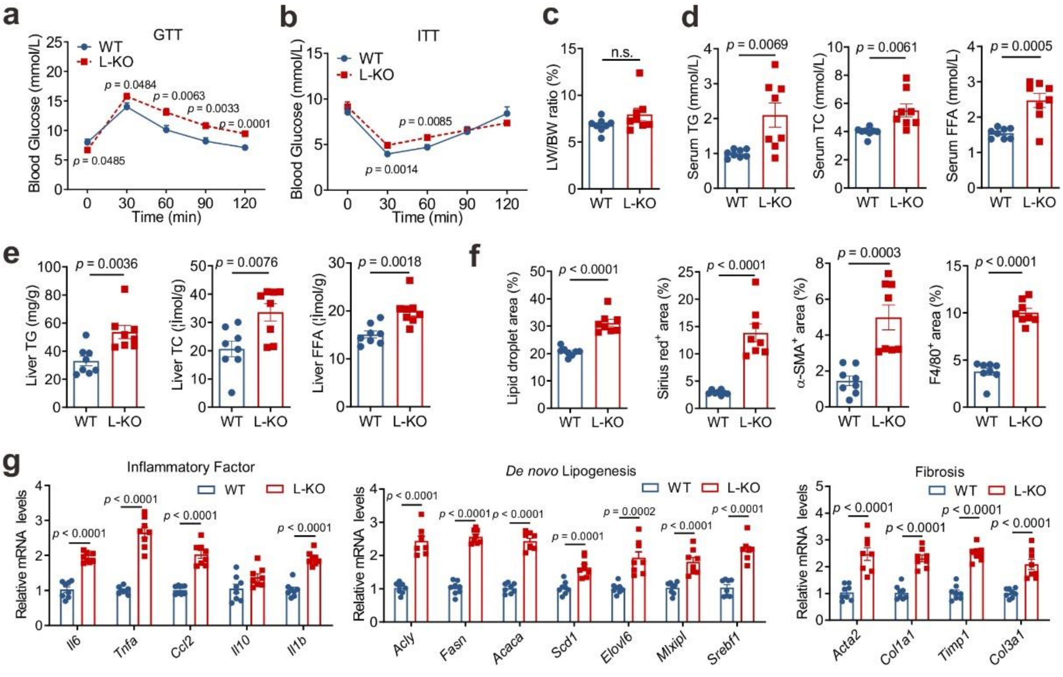

Supplementary Fig. 2 L-KO mice had abnormal lipid metabolism and severe liver injury when fed HFCD-HF/G. For **a**-**g**, WT and L-KO mice were treated with HFCD-HF/G for 24 weeks, for **a**-**b**, n=11/group; for **c**-**g**, n=8/group. **a, b** GTT (**a**) and ITT (**b**) in WT and L-KO mice after 24 weeks of HFCD-HF/G. **c** Liver weight to body weight ratio of mice from the indicated groups. **d, e** TG, TC, and FFA levels in serum (**d**) or liver tissues (**e**). **f** Quantifications of Oil red O staining, Sirius red staining, and IHC staining. **g** Genes associated with inflammatory infiltration, *de novo* lipogenesis, and fibrogenesis were measured in WT and L-KO mice. n was the number of biologically independent mice. Data expressed as mean ± SEM; n.s., not significant. *P* values obtained via 2-tailed unpaired Student's *t* tests. Source data are provided as a Source Data file.

Supplementary Figure 3

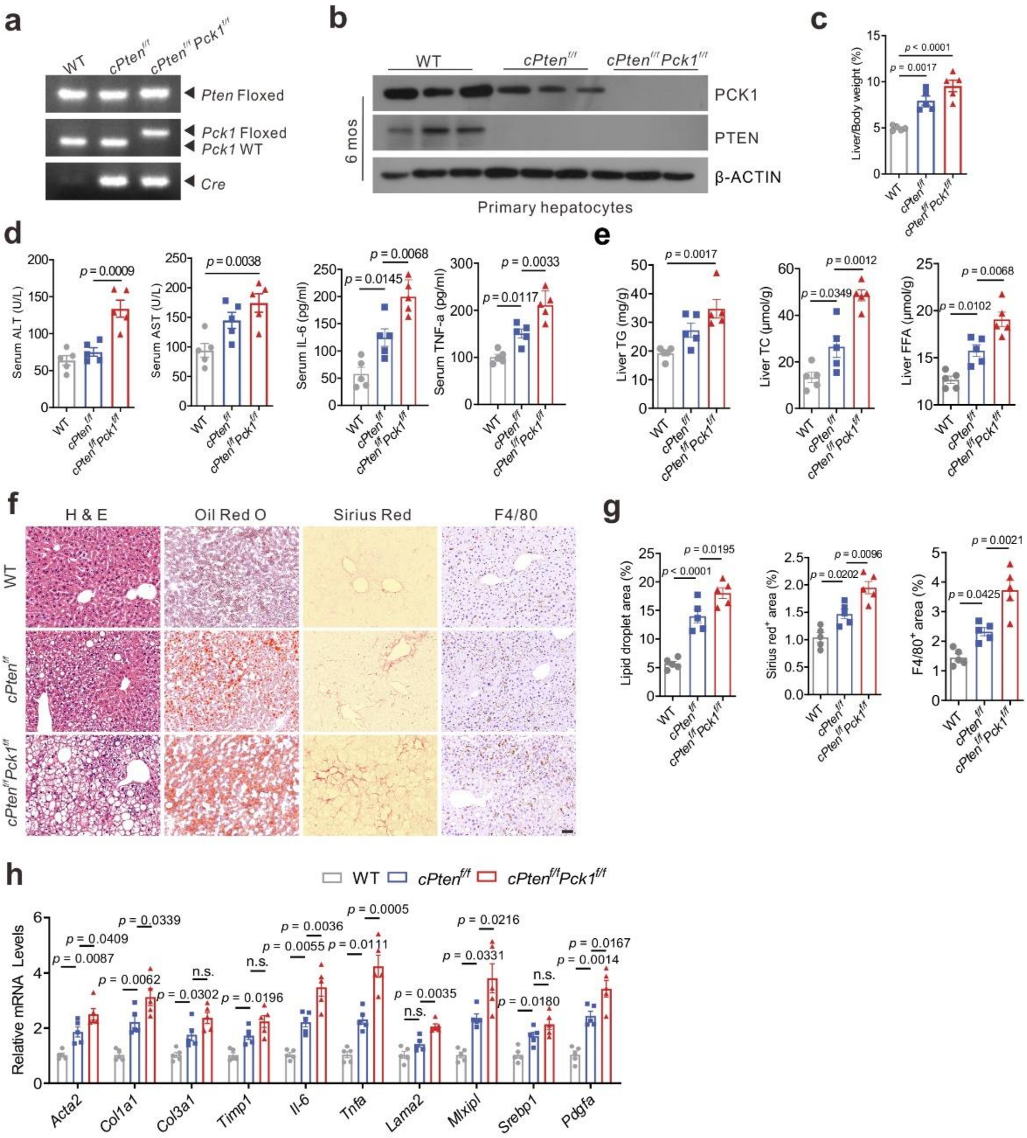

Supplementary Fig. 3 Pck1 depletion accelerates liver steatosis, inflammation and fibrosis in PTEN-null livers. **a** Representative PCR genotyping results from WT, *cPten*$^{f/f}$ and *cPten*$^{f/f}$*Pck1*$^{f/f}$ mice. **b** Western blot analysis of PCK1 and PTEN levels in livers from mice of the indicated genotypes, with β-actin serving as the loading control. **c** Liver weight to body weight (percentage) in three types of mice (n = 5). **d** ALT, AST, IL-6 and TNF-α levels in serum were detected using enzyme-linked immunosorbent assay

(ELISA) (n = 5). **e** Plasma levels of total triglycerides (TG), total cholesterol (TC), and free fatty acids (FFA) (n = 5). **f-g** Paraffin-embedded liver sections were stained with hematoxylin and eosin (H&E), Sirius Red, or immunostained for F4/80. Frozen sections were stained with Oil Red O. Scale bars: 50 μm. **h** Quantitative PCR analysis of liver mRNA expression (n = 5). Data are expressed as the mean ± SEM; n.s., not significant. *P* values obtained via one-way ANOVA with Tukey's post hoc test. Source data are provided as a Source Data file.

Supplementary Figure 4

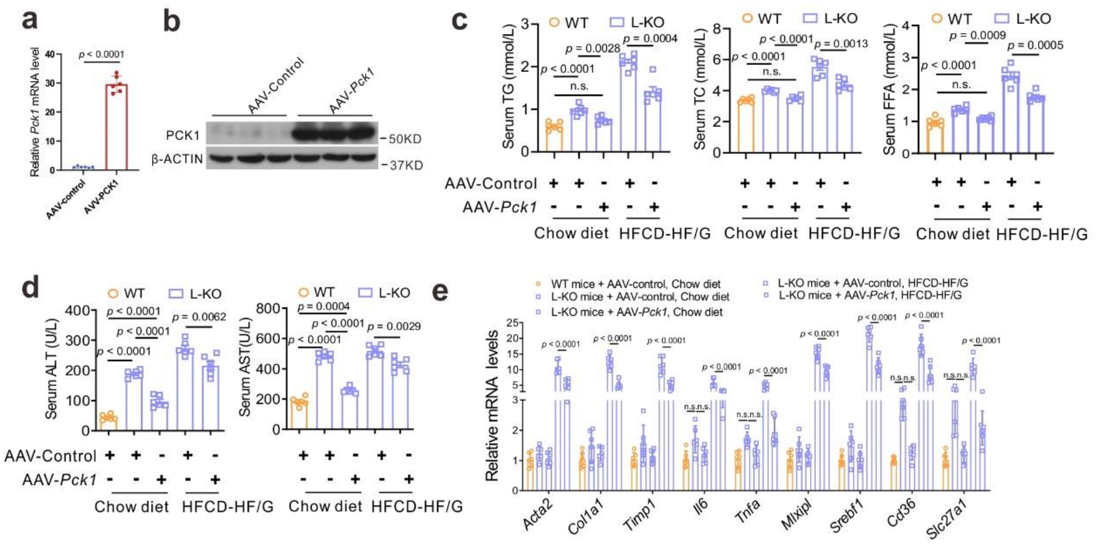

Supplementary Fig. 4 AAV-mediated restoration of hepatic PCK1 alleviates the MAFLD phenotype in *Pck1*-null mice. For **a-e**, mice were administrated with AAV8-TBG-*Control* or AAV8-TBG-*Pck1* after 10 weeks of chow diet or HFCD-HF/G feeding, n=6/group. **a** qPCR analysis and western blot analysis (**b**) of exogenous *Pck1* expression levels in the livers of AAV–injected mice. *P* values obtained via 2-tailed unpaired Student's t tests. The samples were derived from the same experiment and the blots were processed in parallel. **c-d** Levels of serum TG, TC, FFA, ALT and AST concentrations were measured (n = 6). **e** qPCR analysis of genes associated with inflammatory infiltration, *de novo* lipogenesis and fibrogenesis in mice administrated with AAV8-TBG-Control or AAV8-TBG-*Pck1* (n = 6). n was the number of biologically independent mice. Data expressed as mean ± SEM; n.s., not significant. *P* values obtained via one-way ANOVA with Tukey's post hoc test. Source data are provided as a Source Data file.

Supplementary Figure 5

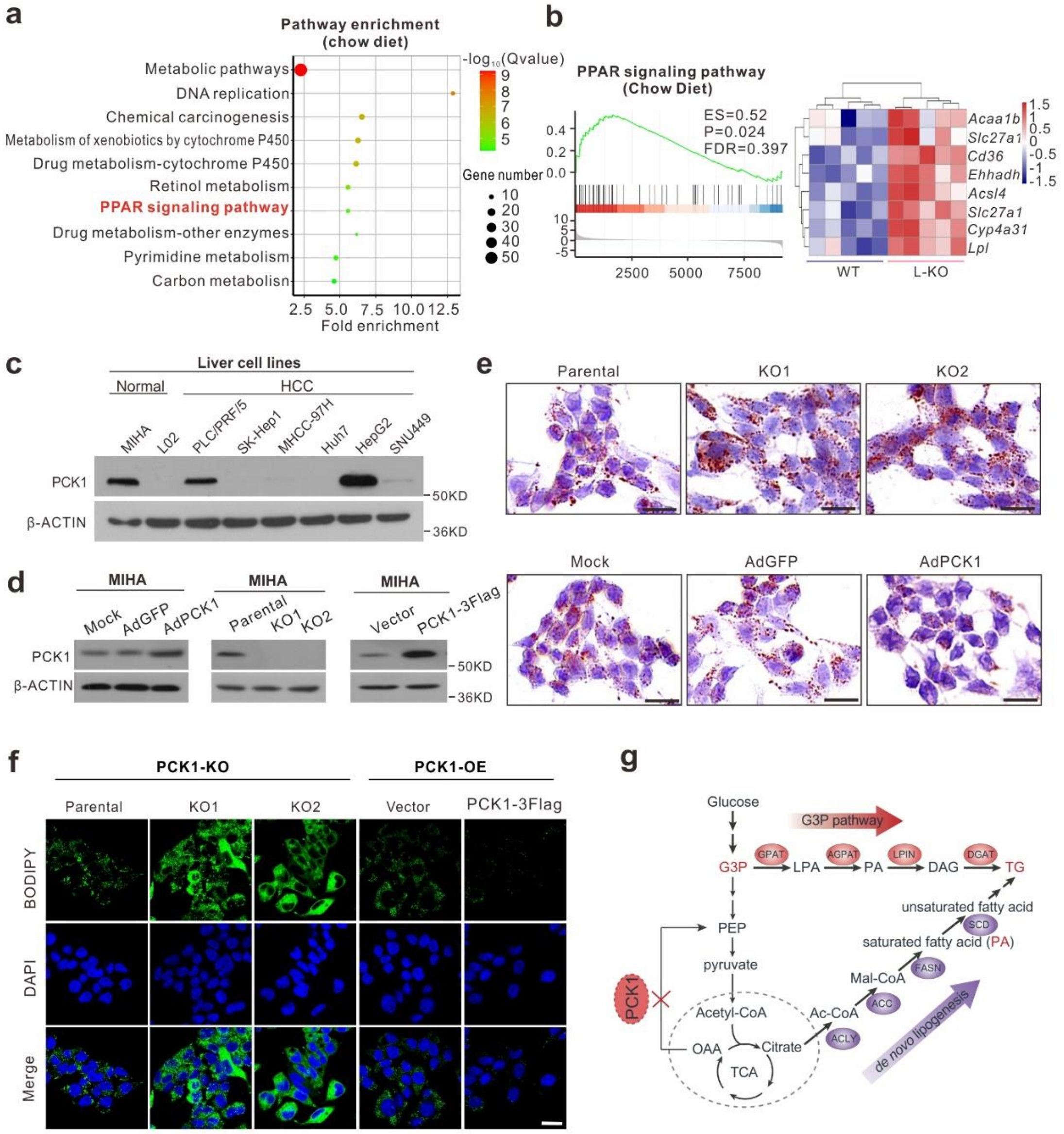

Supplementary Fig. 5 Loss of PCK1 exacerbates lipid metabolism dysfunction *in vitro*. a Pathway enrichment analysis of L-KO mice fed chow diet (n=5). **b** GSEA revealed that the transcription level of PPAR signaling pathway was prominently upregulated in L-KO mice fed chow diet (n=5). **c** Expression levels of PCK1 in different cell lines were measured by western blot. The samples were derived from the same experiment and the blots were processed in parallel. **d** Protein levels of PCK1 were detected by western blot

analysis in *PCK1-OE* and *PCK1-KO* MIHA cells. **e, f** Representative Oil Red O staining (**e**) and BODIPY staining (**f**) in *PCK1-OE* and *PCK1-KO* MIHA cells. Scale bars: 25 μm. The samples were derived from the same experiment. **g** Schematic presentation of the G3P pathway and DNL. G3P: glycerol-3-phosphate; PA: palmitic acid; DL: de novo lipogenesis. n was the number of biologically independent mice. Source data are provided as a Source Data file.

Supplementary Figure 6

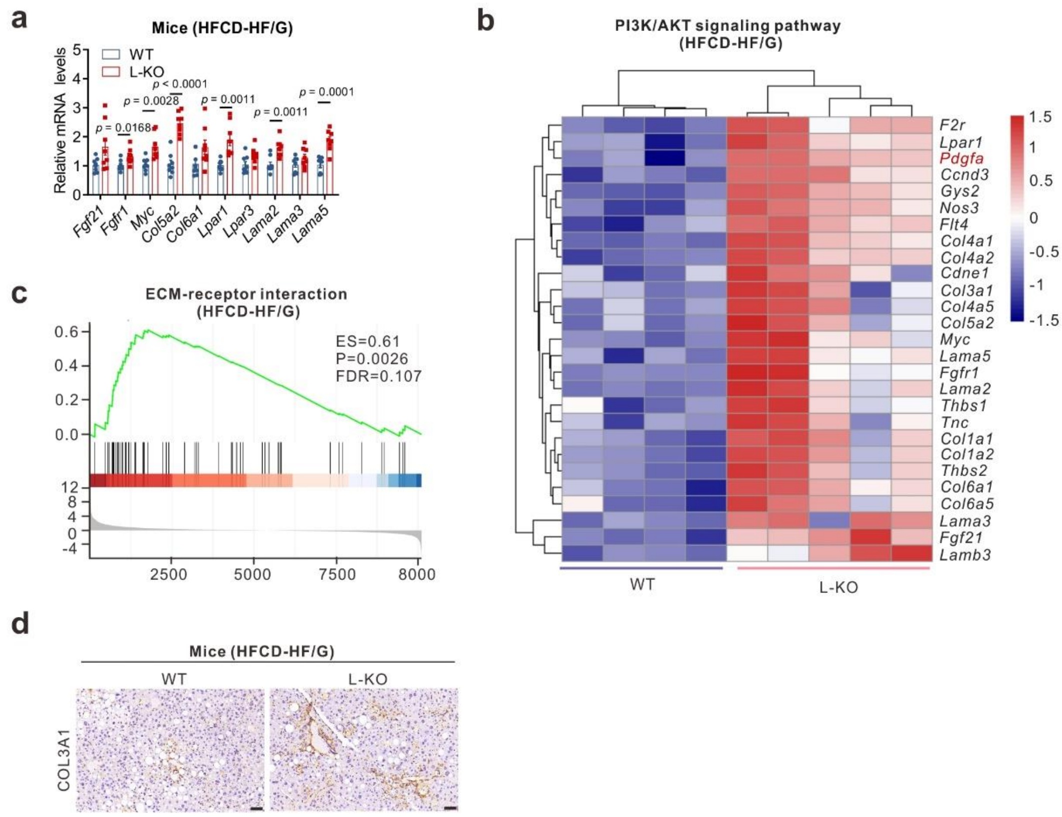

Supplementary Fig. 6 PI3K/AKT signaling pathway and ECM-receptor interaction were activated in L-KO mice fed HFCD-HF/G. a mRNA levels of the indicated genes in the liver of WT and L-KO mice (n = 8). **b** Heatmap of expression levels of PI3K/AKT target genes in WT (n = 4) and L-KO mice (n = 5). **c** GSEA revealed the “ECM-receptor interaction” was upregulated in L-KO mice. **d** COL3A1 immunostaining in mice liver sections. Scale bars: 50 μm. n was the number of biologically independent mice. Data expressed as mean ± SEM; *P* values obtained via 2-tailed unpaired Student’s *t* tests. Source data are provided as a Source Data file.

Supplementary Figure 7

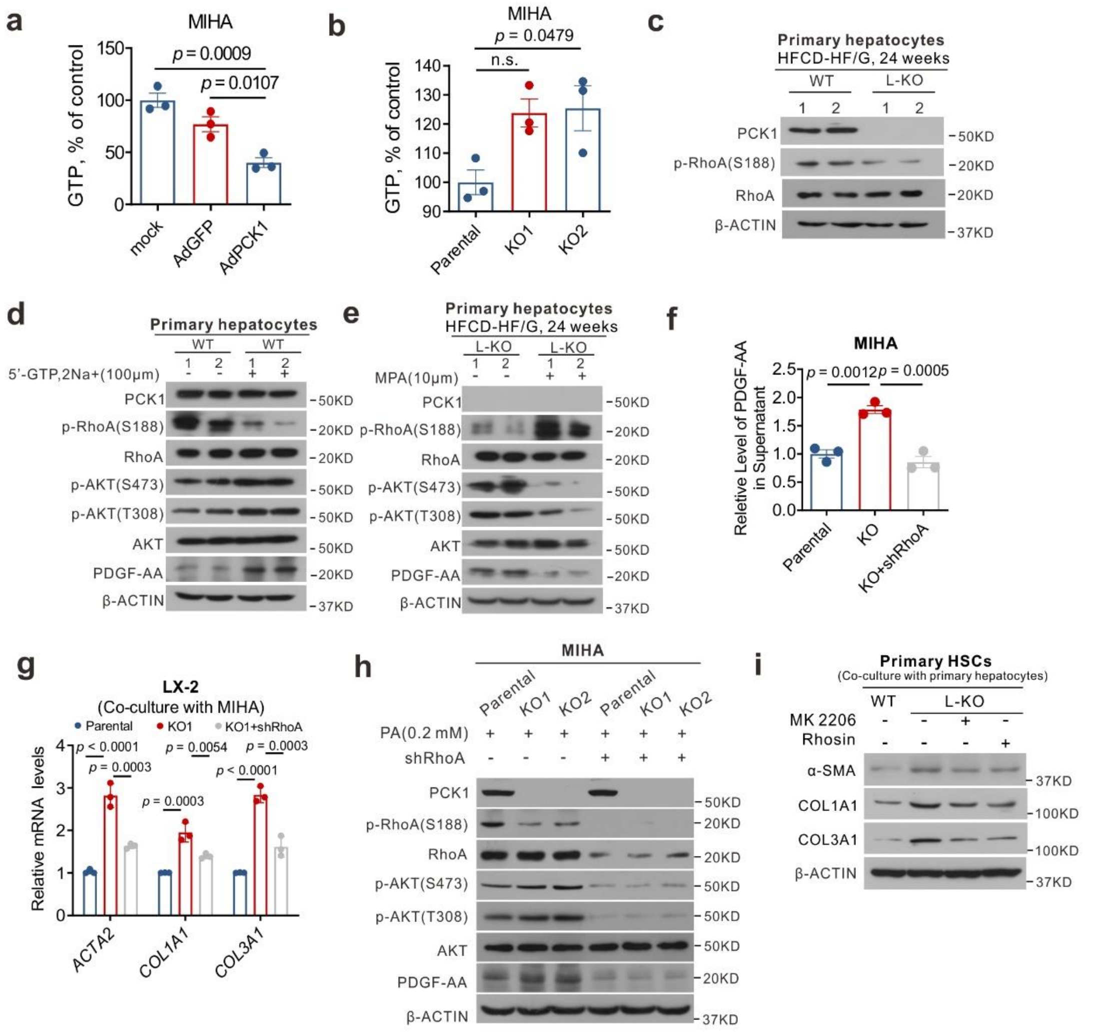

Supplementary Fig. 7 PCK1 deficiency promoted the accumulation of GTP, and RhoA knockdown reversed the activation of PI3K/AKT/PDGF-AA axis.

a-b The intracellular GTP levels in *PCK1*-OE (**a**) and *PCK1*-KO (**b**) cells treated with 0.2 mM PA were determined by HPLC. **c** Immunoblots of the indicated proteins in the primary hepatocytes isolated from WT and L-KO mice fed HFCD-HF/G. **d-e** Immunoblot analysis of AKT, p-AKT (S473 or T308), PDGF-AA, p-RhoA (S188) and RhoA in primary hepatocytes from HFCD-HF/G feeding mice. Cells were treated with 100 μM 5'-GTP, 2Na^{+} for 120 min (**d**) or 10 μM MPA for

48h (**e**). **f** Levels of PDGF-AA in the supernatant of *PCK1*-KO MIHA cells infected with either shControl or shRhoA treated with 0.2 mM PA. **g** Relative mRNA expression of *ACTA2*, *COL1A1*, and *COL3A1* in LX-2 cells co-cultured with *PCK1*-KO MIHA cells infected with either shControl or shRhoA. **h** Immunoblot analysis of indicated proteins in *PCK1*-KO MIHA cells infected with either shControl or shRhoA. **i** Isolated mouse primary hepatocytes from WT and L-KO mice fed HFCD-HF/G for 24 weeks were treated with MK2206, Rhosin or DMSO vehicle, and then co-cultured with primary hepatic stellate cell isolated from WT mice fed chow diet, and the protein levels were determined. For **a**, **b**, **f** and **g**, n = 3. Data expressed as mean ± SEM; n.s., not significant. *P* values obtained via one-way ANOVA with Tukey's post hoc test. Source data are provided as a Source Data file.

Supplementary Figure 8

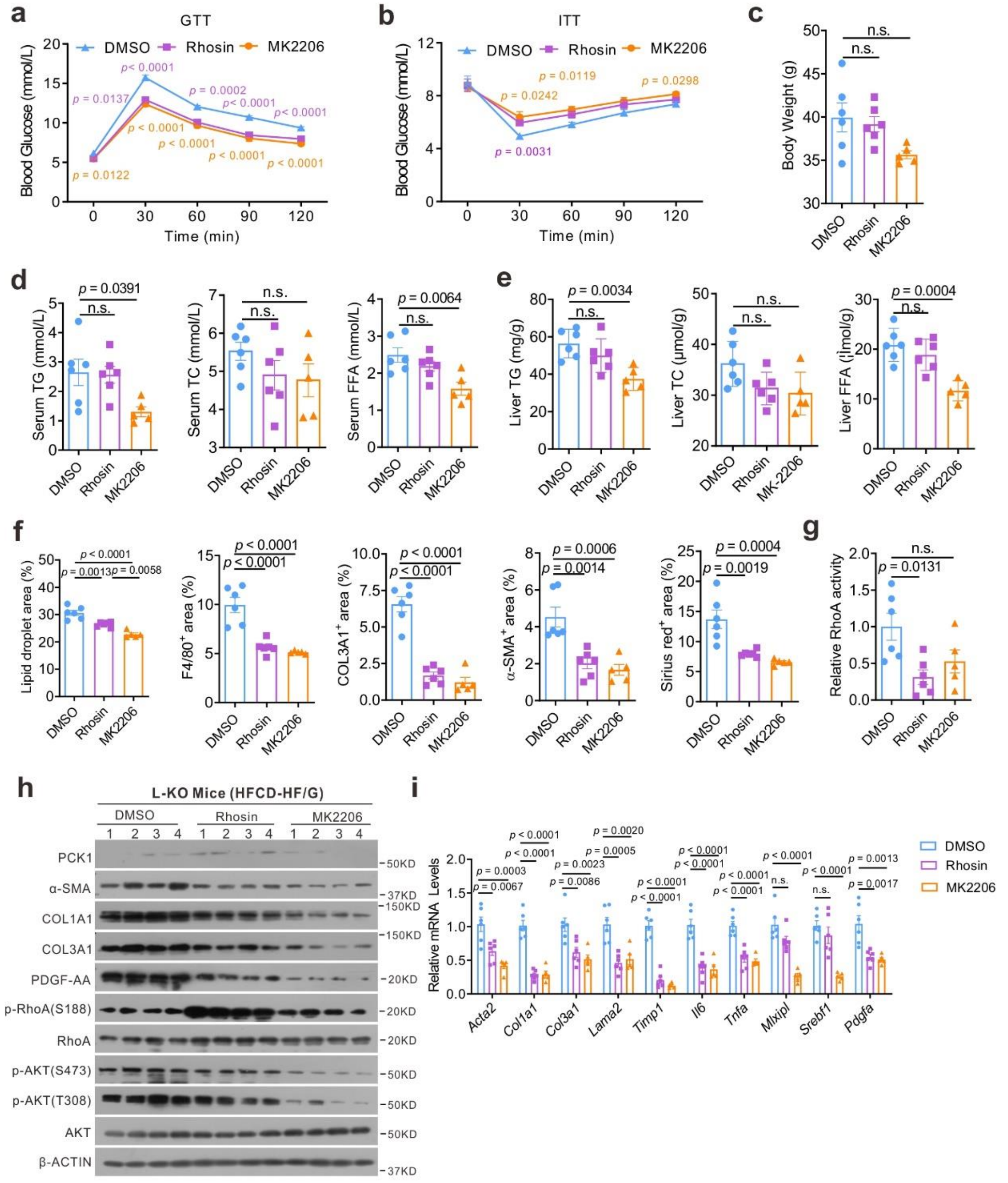

Supplementary Fig. 8 RhoA/AKT inhibition partially relieves MAFLD phenotype in L-KO mice fed HFCD-HF/G. a, b Glucose levels measured during the glucose tolerance test (GTT) (**a**) and insulin tolerance test (ITT) (**b**). **c** Body weight of mice in the indicated groups. **d** Serum TG, TC, and FFA levels were measured using automated biochemical analyzer. **e** Liver TG, TC, and

FFA levels determined by colorimetry. **f** Quantification of liver sections of L-KO mice treated with DMSO, Rhosin or MK2206. **g** Reletive GTP-bound RhoA levels in mice liver tissues. **h** Expression of the indicated proteins in mice liver tissues. **i** mRNA levels of genes associated with lipid metabolism, fibrogenesis, and inflammatory infiltration. DMSO group (n = 6), Rhosin group (n = 6), MK2206 group (n = 5). n was the number of biologically independent mice. Data expressed as mean ± SEM; n.s., not statistically significant. *P* values obtained via one-way ANOVA with Tukey's post hoc test. Source data are provided as a Source Data file.

Supplementary Figure 9

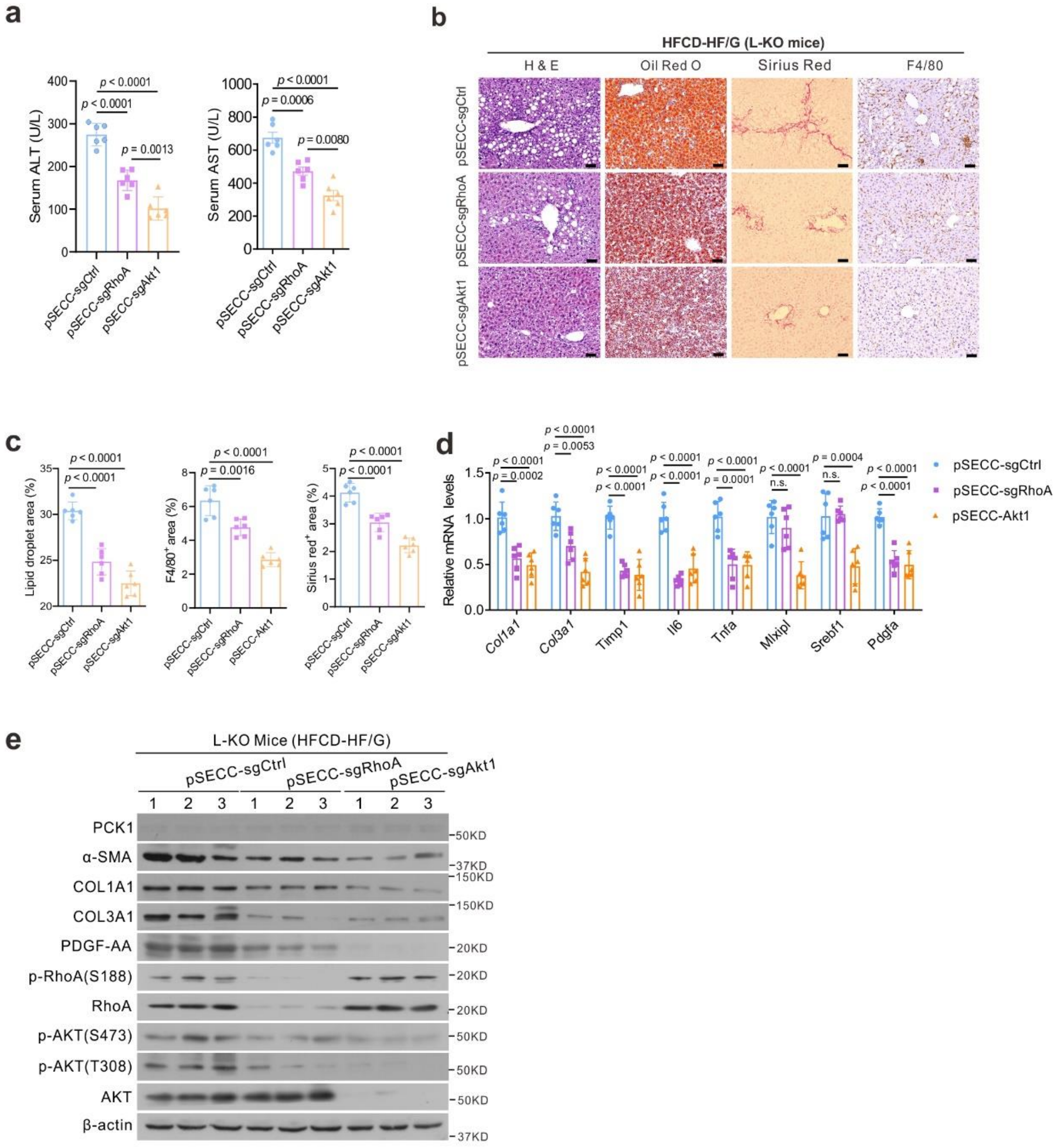

Supplementary Fig. 9 Genetic inhibition of RhoA or AKT1 protects mice from diet-induced MAFLD pathologies in L-KO mice. a Levels of AST and ALT in plasma (n = 6). **b** Representative HE staining, Oil Red O staining and immunohistochemical staining of liver tissues from L-KO mice infected with the control pSECC-sgTom lentivirus, the pSECC-sgAKT1 or the pSECC-sgRhoA lentivirus. Scale bars: 50 μm. **c** Quantification of the Oil Red O and immunohistochemical staining (n = 6). **d** Relative mRNA expression in liver

tissue of various groups was analyzed by qPCR (n = 6). **e** Western blot analysis of indicated protein expression in mice liver tissues. n was the number of biologically independent mice. Data expressed as mean ± SEM; not statistically significant. *P* values obtained via one-way ANOVA with Tukey's post hoc test. Source data are provided as a Source Data file.

Supplementary Tables

Supplementary Table 1. Clinical characteristics of participants with control and NASH patients.

	Control	NASH	*P* value
N (male/female)	10(5/5)	36(18/18)	NS
Age (years)	43.7 ± 4.94	44.64 ± 2.81	NS
BMI (kg/m^2)	21.34 ± 0.92	27.54 ± 0.76	<0.0001
ALT (U/L)	21.5 ± 4.54	136.2 ± 11.47	<0.0001
AST (U/L)	23.4 ± 1.56	74 ± 6.52	0.0002
γ-GT (U/L)	18.6 ± 1.69	83.17 ± 10.49	0.0025
TC (mmol/L)	2.30 ± 0.20	4.56 ± 0.20	<0.0001
TG (mmol/L)	1.09 ± 0.15	1.41 ± 0.12	NS
Glucose (mmol/L)	4.71 ± 0.17	7.79 ± 0.38	0.0001

Clinical and biochemical characteristics of NASH patients and controls. Data are expressed as mean ± SEM. Differences were analyzed by the two-tailed unpaired *t* test. BMI: body mass index; ALT: alanine transaminase; AST: aspartate transaminase; γ-GT: γ-glutamyl-transferase; TC: total cholesterol; TG: triglyceride.

Supplementary Table 2. Information on reagents.

**Name**	**Supplier**	**Cat no.**
MK2206	Selleckchem	S1078
Rhosin	MCE	HY-12646
Palmatic acid	Sigma Aldrich	P0500
BSA	Solarbio	A8020-100G
pAdTrack-TO4	Dr.T-C He, University of Chicago	N/A
pSEB-3Flag	Dr.T-C He, University of Chicago	N/A
AdEasy-BJ5183 E. coli	Dr.T-C He, University of Chicago	N/A
DH10B Chemically Competent E. coli	Dr.T-C He, University of Chicago	N/A
pReceiver-M02-ATF3	GeneCopoeia	EX-F0674-M02
AdGFP	Dr.T-C He, University of Chicago	N/A
AdPCK1	Lab stock[1]	N/A
pSEB-3Flag - PCK1	Lab stock[1]	N/A
LentiCRISPER-V2	Prof. Ding Xue, Tsinghua University	N/A
LentiCRISPER-*PCK1* KO1	Lab stock[1]	N/A
LentiCRISPER-*PCK1* KO2	Lab stock[1]	N/A

Supplementary Table 3. Primer sequences used in this study.

**Name**	**Sequence**	**Supplier**
sub-clone primer: *PCK1*-Forward	CGCGGATCCACCATGGGCCCTCCTCAGCTGCAAAACGGCC	TsingKe Biological Technology, China
sub-clone primer: *PCK1*-Reverse	CCCAAGCTTCTACATCTGGCTTATTCTTTGCTTCAAG	TsingKe Biological Technology, China
real-time PCR: *PCK1*-Forward	ATGGAGGAAGAGGGCATCCT	TsingKe Biological Technology, China
real-time PCR: *PCK1*-Reverse	ACGTACATGGTGCGACCTTT	TsingKe Biological Technology, China
real-time PCR: *β-ACTIN*-Forward	AGGCCAACCGCGAGAAGATGACC	TsingKe Biological Technology, China
real-time PCR: *β-ACTIN*-Reverse	GAAGTCCAGGGCGACGTAGCAC	TsingKe Biological Technology, China
real-time PCR: *ACTA2*-Forward	GGGGTGATGGTGGGAATG	TsingKe Biological Technology, China
real-time PCR: *ACTA2*-Reverse	GCAGGGTGGGATGCTCTT	TsingKe Biological Technology, China
real-time PCR: *COL1A1*-	GACGGCTCAGAGTCACCCA	TsingKe Biological Technology, China

Forward		
real-time PCR: *COL1A1*-Reverse	GGAGACCACGAGGACCAGA	TsingKe Biological Technology, China
real-time PCR: *COL3A1*-Forward	GCTCGGGGTAATGACGGT	TsingKe Biological Technology, China
real-time PCR: *COL3A1*-Reverse	AGGAATGCCAGCGGGAC	TsingKe Biological Technology, China
real-time PCR: *PDGFA*-Forward	TGTCAAGTGCCAGCCCTCC	TsingKe Biological Technology, China
real-time PCR: *PDGFA*-Reverse	CCGTGTCCTCTTCCCGATAAT	TsingKe Biological Technology, China
real-time PCR: *TIMP1*-Forward	GCTTCTGGCATCCTGTTGTT	TsingKe Biological Technology, China
real-time PCR: *TIMP1*-Reverse	TGGTTGACTTCTGGTGTCCC	TsingKe Biological Technology, China
real-time PCR: *SERPINH1*-Forward	GCCATGTTCTTCAAGCCACA	TsingKe Biological Technology, China
real-time PCR:	CTTTTCCTTCTCGTCGTCGTA	TsingKe Biological

SERPINH1-Reverse		Technology, China
real-time PCR: *GFAP*-Forward	GCACGCAGTATGAGGCAATG	TsingKe Biological Technology, China
real-time PCR: *GFAP*-Reverse	CCAGGTCGCAGGTCAAGGA	TsingKe Biological Technology, China
real-time PCR: *MMP2*-Forward	TTTGACGGTAAGGACGGACTC	TsingKe Biological Technology, China
real-time PCR: *MMP2*-Reverse	CCTGGAAGCGGAATGGAAAC	TsingKe Biological Technology, China
real-time PCR: *VIM*-Forward	GAGAACTTTGCCGTTGAAGC	TsingKe Biological Technology, China
real-time PCR: *VIM*-Reverse	TCCAGCAGCTTCCTGTAGGT	TsingKe Biological Technology, China
real-time PCR: *PGC1A*-Forward	GATGGCCTGTTTGATGACAG	TsingKe Biological Technology, China
real-time PCR: *PGC1A*-Reverse	TTTGGGTGGTGACACAGAAT	TsingKe Biological Technology, China
real-time PCR: *FOXO1*-Forward	TGTCAACCTATGGCAGCCAG	TsingKe Biological Technology, China
real-time PCR: *FOXO1*-Reverse	GCAGAGGCACTTGTACAGGT	TsingKe Biological Technology, China

real-time PCR: *ATF3*-Forward	TGAGTGCTTCTGCCATCGTC	TsingKe Biological Technology, China
real-time PCR: *ATF3*-Reverse	GGCTACCTCGGCTTTTGTG	TsingKe Biological Technology, China
real-time PCR: *HNF4A*-Forward	GCCTACCTCAAAGCCATCAT	TsingKe Biological Technology, China
real-time PCR: *HNF4A*-Reverse	CGGTCGTTGATGTAGTCCTC	TsingKe Biological Technology, China
real-time PCR: *CEBPB*-Forward	TCGCAGGTCAAGAGCAAGG	TsingKe Biological Technology, China
real-time PCR: *CEBPB*-Reverse	GAACAAGTTCCGCAGGGTG	TsingKe Biological Technology, China
real-time PCR: *SREBF1*-Forward	TCTGGAGGCATCGCAAGC	TsingKe Biological Technology, China
real-time PCR: *SREBF1*-Reverse	CAGCAGGTGACGGATGAGG	TsingKe Biological Technology, China
real-time PCR: *NR1H3*-Forward	GGTACAACCCTGGGAGTGAGA	TsingKe Biological Technology, China
real-time PCR: *NR1H3*-Reverse	TGGGGATGGTGGATGGAG	TsingKe Biological Technology, China

real-time PCR: *MLXIPL*-Forward	GTCGGCAATGCTGACATGA	TsingKe Biological Technology, China
real-time PCR: *MLXIPL*-Reverse	GCTGAAGAGGGAGTCAACCAC	TsingKe Biological Technology, China
real-time PCR: *NR1H4*-Forward	AACTCACCCCAGATCAACAGAC	TsingKe Biological Technology, China
real-time PCR: *NR1H4*-Reverse	GCTTCAACCGCAGACCCT	TsingKe Biological Technology, China
real-time PCR: *Il6*-Forward	GTTGTGCAATGGCAATTCTGA	TsingKe Biological Technology, China
real-time PCR: *Il6*- Reverse	AAGGACTCTGGCTTTGTCTTTCT	TsingKe Biological Technology, China
real-time PCR: *Tnfα*-Forward	CCTGCCCCAAGGACACC	TsingKe Biological Technology, China
real-time PCR: *Tnfα*-Reverse	AGAGCAATGACTCCAAAGTAGACC	TsingKe Biological Technology, China
real-time PCR: *Il1b*-Forward	AAGCCTCGTGCTGTCGGA	TsingKe Biological Technology, China
real-time PCR: *Il1b* - Reverse	CCATCTTCTTCTTTGGGTATTGC	TsingKe Biological Technology, China
real-time PCR: *Il10*-Forward	GGTTGCCAAGCCTTATCGG	TsingKe Biological Technology, China

real-time PCR: *Il10*-Reverse	ATTTTCACAGGGGAGAAATCG	TsingKe Biological Technology, China
real-time PCR: *Ccl2*-Forward	TGTGCTGACCCCAAGAAGG	TsingKe Biological Technology, China
real-time PCR: *Ccl2*- Reverse	GGTGGTTGTGGAAAAGGTAGTG	TsingKe Biological Technology, China
real-time PCR: *Acta2*-Forward	CCCTGAAGAGCATCCGACA	TsingKe Biological Technology, China
real-time PCR: *Acta2*- Reverse	CATCTCCAGAGTCCAGCACAA	TsingKe Biological Technology, China
real-time PCR: *Col1a1*-Forward	ACCCTGCCCGCACATG	TsingKe Biological Technology, China
real-time PCR: *Col1a1*- Reverse	CCCTCGCTTCCGTACTCG	TsingKe Biological Technology, China
real-time PCR: *Lama2*-Forward	TCCAGCCAAACCATCAGTCC	TsingKe Biological Technology, China
real-time PCR: *Lama2*- Reverse	CCACAAGAAGGTCCAATCCAAC	TsingKe Biological Technology, China
real-time PCR: *Timp1*-Forward	CCCAGAAATCAACGAGACCA	TsingKe Biological Technology, China
real-time PCR: *Timp1*-Reverse	ACGCCAGGGAACCAAGAA	TsingKe Biological Technology, China

real-time PCR: *Col3a1*-Forward	GCCCACAGCCTTCTACACCT	TsingKe Biological Technology, China
real-time PCR: *Col3a1*-Reverse	TCCCGGATAGCCACCCA	TsingKe Biological Technology, China
real-time PCR: *Acly*-Forward	CTCATTGAACCCTTCGTCCC	TsingKe Biological Technology, China
real-time PCR: *Acly* - Reverse	CCTCGGTATTCAGCTTTTCGT	TsingKe Biological Technology, China
real-time PCR: *Fasn*-Forward	CTGCCTCCGTGGACCTTATC	TsingKe Biological Technology, China
real-time PCR: *Fasn* - Reverse	GCACAGACACCTTCCCGTCA	TsingKe Biological Technology, China
real-time PCR: *Acaca*–Forward	ATTGCCTATGAACTCAACAGCG	TsingKe Biological Technology, China
real-time PCR: *Acaca* - Reverse	TGACAAGGTGGCGTGAAGG	TsingKe Biological Technology, China
real-time PCR: *Scd1*-Forward	TTCCTTATCATTGCCAACACCA	TsingKe Biological Technology, China
real-time PCR: *Scd1*- Reverse	TCGCCCCAGCAGTACCAG	TsingKe Biological Technology, China
real-time PCR: *Elovl6*-Forward	TGCGGGCTGCGGGTTT	TsingKe Biological Technology, China

real-time PCR: *Elovl6*- Reverse	GCCTTCGTGGCTTTCTTCACT	TsingKe Biological Technology, China
real-time PCR: *Cpt1a*-Forward	TGTCCAAGTATCTGGCAGTCG	TsingKe Biological Technology, China
real-time PCR: *Cpt1a* - Reverse	CATAGCCGTCATCAGCAACC	TsingKe Biological Technology, China
real-time PCR: *Mlxipl*-Forward	GCTGCGGGATGAAATAGAGG	TsingKe Biological Technology, China
real-time PCR: *Mlxipl*-Reverse	TCAAATAAAGGTCGGATGAGGA	TsingKe Biological Technology, China
real-time PCR: Srebf1-Forward	TTCTGGAGACATCGCAAACAA	TsingKe Biological Technology, China
real-time PCR: Srebf1- Reverse	TGGTAGACAACAGCCGCATC	TsingKe Biological Technology, China
real-time PCR: *Ppara*-Forward	GACATTTCCCTGTTTGTGGCT	TsingKe Biological Technology, China
real-time PCR: *Ppara*-Reverse	GCTGCGTCGGACTCGGT	TsingKe Biological Technology, China
real-time PCR: *Cd36*-Forward	ACTGTGGGCTCATTGCTGG	TsingKe Biological Technology, China
real-time PCR: *Cd36*-Reverse	TGATTTTGCTGCTGTTCTTTGC	TsingKe Biological Technology, China

real-time PCR: *Slc27a1*-Forward	GCTCCTGCGGCTTCAACA	TsingKe Biological Technology, China
real-time PCR: *Slc27a1*-Reverse	GCGCTATCGCCCTTTCG	TsingKe Biological Technology, China
real-time PCR: *Cidea*-Forward	CCTGGTTACGCTGGTGCTG	TsingKe Biological Technology, China
real-time PCR: *Cidea*-Reverse	TGGCTATTCCCGATTTCTTTG	TsingKe Biological Technology, China
real-time PCR: *Cidec*-Forward	AAGGTTCGCAAAGGCATCA	TsingKe Biological Technology, China
real-time PCR: *Cidec*-Reverse	GGCTTCTGGGAAAGGGCTA	TsingKe Biological Technology, China
real-time PCR: *Yap1*-Forward	TTTCGGCAGGCAATACGG	TsingKe Biological Technology, China
real-time PCR: *Yap1*-Reverse	GGGTGCTTTGGCTGATGGT	TsingKe Biological Technology, China
real-time PCR: *Ctgf*-Forward	TTGGCCCAGACCCAACTA	TsingKe Biological Technology, China
real-time PCR: *Ctgf* -Reverse	GCAGGAGGCGTTGTCATT	TsingKe Biological Technology, China
real-time PCR: *Gli1*-Forward	CGTTTGAAGGCTGTCGGAAGT	TsingKe Biological Technology, China

real-time PCR: *Gli1*-Reverse	GCGGAGCGAGCTGGGAT	TsingKe Biological Technology, China
real-time PCR: *Tgfb1*-Forward	CCGCAACAACGCCATCTA	TsingKe Biological Technology, China
real-time PCR: *Tgfb1*-Reverse	ACTGCCGTACAACTCCAGTGAC	TsingKe Biological Technology, China
real-time PCR: *Pdgfa*-Forward	TGTAACACCAGCAGCGTCAA	TsingKe Biological Technology, China
real-time PCR: *Pdgfa*-Reverse	CCTTCCTGTCTCCTCCTCCC	TsingKe Biological Technology, China
real-time PCR: *Igf1*-Forward	GGACCGAGGGGCTTTTACT	TsingKe Biological Technology, China
real-time PCR: *Igf1*-Reverse	ATAGAGCGGGCTGCTTTTG	TsingKe Biological Technology, China
real-time PCR: Vegfa-Forward	CTACTGCCGTCCGATTGAGA	TsingKe Biological Technology, China
real-time PCR: *Vegfa*-Reverse	CTGGCTTTGGTGAGGTTTGAT	TsingKe Biological Technology, China
real-time PCR: *Fgf21*-Forward	GCATACCCCATCCCTGACTC	TsingKe Biological Technology, China
real-time PCR: *Fgf21*-Reverse	GGCTGTTGGCAAAGAAACCTA	TsingKe Biological Technology, China

real-time PCR: *Fgfr1*-Forward	GGATTCTGTGGTGCCTTCTGA	TsingKe Biological Technology, China
real-time PCR: *Fgfr1*-Reverse	TTGTCTGGCCCGATCTTACTC	TsingKe Biological Technology, China
real-time PCR: *Myc*-Forward	GACTGTATGTGGAGCGGTTTCT	TsingKe Biological Technology, China
real-time PCR: *Myc*-Reverse	TCGTTGAGCGGGTAGGGA	TsingKe Biological Technology, China
real-time PCR: *Col5a2*-Forward	TGTGCGGGGCAGTGTAGG	TsingKe Biological Technology, China
real-time PCR: *Col5a2*-Reverse	TCCCAGGGTCTGTTTTGTTTG	TsingKe Biological Technology, China
real-time PCR: *Col6a1*-Forward	GGGGTCAAAGGGGCAAAG	TsingKe Biological Technology, China
real-time PCR: *Col6a1*-Reverse	GGCAATCTCAAAGTTCTGTAGGC	TsingKe Biological Technology, China
real-time PCR: *Lpar1*-Forward	TCCATACACGAATGAGCAACC	TsingKe Biological Technology, China
real-time PCR: *Lpar1*-Reverse	TGGCGAACATAGCCAAAGAT	TsingKe Biological Technology, China
real-time PCR: *Lpar3*-Forward	CTGCTCGCACTGCTCAACTC	TsingKe Biological Technology, China

real-time PCR: *Lpar3*-Reverse	CTGGCTGCCCGTCTCG	TsingKe Biological Technology, China
real-time PCR: *Gpat3*-Forward	CTTCCAGACAGCAGCCTCAA	TsingKe Biological Technology, China
real-time PCR: *Gpat3*-Reverse	TCCCCATCAATCCACCGT	TsingKe Biological Technology, China
real-time PCR: *Gpat4*-Forward	CAAGCCCTACACCAACGGAA	TsingKe Biological Technology, China
real-time PCR: *Gpat4*-Reverse	TGGCAGGAGGAAGCAATACC	TsingKe Biological Technology, China
real-time PCR: *Agpat2*-Forward	CTGCTGTTGCTGCTTGTGC	TsingKe Biological Technology, China
real-time PCR: *Agpat2*-Reverse	CCTCCAGTTTCTTCTGTCCG	TsingKe Biological Technology, China
real-time PCR: *Agpat3*-Forward	GCTTGCCTACCTGAAGACCC	TsingKe Biological Technology, China
real-time PCR: *Agpat3*-Reverse	CCAAACCGCTCGCACATC	TsingKe Biological Technology, China
real-time PCR: *Lpin1*-Forward	TGCTCATCCACCAGAGTAAGG	TsingKe Biological Technology, China
real-time PCR: *Lpin1*-Reverse	TCCGTGAGGTCGTCCAGAT	TsingKe Biological Technology, China

real-time PCR: *Lpin2*-Forward	CCCCTCCTGGGATTCTGTC	TsingKe Biological Technology, China
real-time PCR: *Lpin2*-Reverse	TGAAAAGGCGAGCACTGGTA	TsingKe Biological Technology, China
real-time PCR: *Lpin3*-Forward	GGATGACCCAAACCTCGTG	TsingKe Biological Technology, China
real-time PCR: *Lpin3*-Reverse	TTGCGGCTTTCTCCCTCT	TsingKe Biological Technology, China
real-time PCR: *Dgat1*-Forward	AAGACGGGCGGACCAGC	TsingKe Biological Technology, China
real-time PCR: *Dgat1*-Reverse	CACCAGGATGCCATACTTGATA	TsingKe Biological Technology, China
real-time PCR: *Dgat2*-Forward	CTGCGGGGTGAGCGTC	TsingKe Biological Technology, China
real-time PCR: *Dgat2*-Reverse	ACCTTTCTTGGGCGTGTTC	TsingKe Biological Technology, China
real-time PCR: *Mmp1*-Forward	GGCTGAAAGTGACTGGGAAAC	TsingKe Biological Technology, China
real-time PCR: *Mmp1*-Reverse	TGGCAAATCTGGCGTGTAA	TsingKe Biological Technology, China
real-time PCR: *Mmp9*-Forward	GCCCTGAACCTGAGCCA	TsingKe Biological Technology, China

real-time PCR: *Mmp9*-Reverse	ACTTCCCATCCTTGAACAAATAC	TsingKe Biological Technology, China
real-time PCR: *Lama2*-Forward	TCCAGCCAAACCATCAGTCC	TsingKe Biological Technology, China
real-time PCR: *Lama2*-Reverse	CCACAAGAAGGTCCAATCCAAC	TsingKe Biological Technology, China
real-time PCR: *Lama3*-Forward	CCGCTCGGGCTCCTATT	TsingKe Biological Technology, China
real-time PCR: *Lama3*-Reverse	ACATGCTGCTTGCACTGACA	TsingKe Biological Technology, China
real-time PCR: *Lama5*-Forward	GGAATATGTCGGTGAGGATTCA	TsingKe Biological Technology, China
real-time PCR: *Lama5*-Reverse	TCCAGGTAGAAGATGGCTAGATG	TsingKe Biological Technology, China
real-time PCR: *β-Actin*-Forward	CGTTCAATACCCCAGCCATG	TsingKe Biological Technology, China
real-time PCR: *β-Actin*-Reverse	GACCCCGTCACCAGAGTCC	TsingKe Biological Technology, China
ChIP qPCR: *PCK1*-Forward 1	CCCAAAGCATAACTGACCCTG	TsingKe Biological Technology, China
ChIP qPCR: *PCK1*-Reverse 1	TTAAATACTGTGGAAAAGAATAGCC	TsingKe Biological Technology, China

ChIP qPCR: *PCK1*-Forward 2	TGGTTGAGGGCTCGAAGTC	TsingKe Biological Technology, China
ChIP qPCR: *PCK1*-Reverse 2	ACGGCCAGGGTCAGTTATG	TsingKe Biological Technology, China
ChIP qPCR: *PCK1*-Forward 3	CCCAGCATTCATTAACAACTATCT	TsingKe Biological Technology, China
ChIP qPCR: *PCK1*-Reverse 3	TGCTTGGTGGCAGAACCTC	TsingKe Biological Technology, China
sgRNA: *PCK1*-Forward	CACCGGCTGAAGAAGTATGACAAC	TsingKe Biological Technology, China
sgRNA: *PCK1*-Reverse	AAACGTTGTCATACTTCTTCAGCC	TsingKe Biological Technology, China
TA cloning: *PCK1*-Forward	AACCTGTGGATCTCCCTTC	TsingKe Biological Technology, China
TA cloning: *PCK1*-Reverse	CAAATCAATGTTCCGCTCA	TsingKe Biological Technology, China
shRNA: *ATF3* -Forward 1	TGCAAAGTGCCGAAACAAGATTCAAGAGATCTT GTTTCGGCACTTTGCTTTTTTC	TsingKe Biological Technology, China
shRNA: *ATF3* -Reverse 1	TCGAGAAAAAAGCAAAGTGCCGAAACAAGATC TCTTGAATCTTGTTTCGGCACTTTGCA	TsingKe Biological Technology, China

shRNA: *ATF3* -Forward 2	TGAGAAACCTCTTTATCCAATTCAAGAGATTGG ATAAAGAGGTTTCTCTTTTTTC	TsingKe Biological Technology, China
shRNA: *ATF3* -Reverse 2	TCGAGAAAAAAGAGAAACCTCTTTATCCAATCT CTTGAATTGGATAAAGAGGTTTCTCA	TsingKe Biological Technology, China
shRNA: *ATF3* -Forward 3	TGGACTCCAGAAGATGAGAGTTCAAGAGACTC TCATCTTCTGGAGTCCTTTTTTC	TsingKe Biological Technology, China
shRNA: *ATF3* -Reverse 3	TCGAGAAAAAAGGACTCCAGAAGATGAGAGTC TCTTGAACTCTCATCTTCTGGAGTCCA	TsingKe Biological Technology, China
Genotyping of *Pck1*-Forward	TCTGTCAGTTCAATACCAATCT	TsingKe Biological Technology, China
Genotyping of *Pck1*-Reverse	AATGTTCTCTGCAAGTCCTGGTG	TsingKe Biological Technology, China
Genotyping of *Pten*-Forward	ATCCCCACCAATGAACAAAC	TsingKe Biological Technology, China
Genotyping of *Pten*-Reverse	CTCCTCTACTCCATTCTTCCC	TsingKe Biological Technology, China
Genotyping of *Cre*-Forward	CACCCTGTTACGTATAGCCG	TsingKe Biological Technology, China

Genotyping of *Cre*-Reverse	GAGTCATCCTTAGCGCCGTA	TsingKe Biological Technology, China

Supplementary Table 4. The antibody information.

Name	Supplier	Cat no.	Clone no.	Dilution
PCK1	Bioworld Technology, USA	BS6870	Polyclonal	1:2000
PPARα	Proteintech, USA	15540-1-AP	Polyclonal	1:1000
CD36	Abcam, USA	Ab133625	EPR6573	1:1000
FATP1	Affinity Biosciences, USA	DF7716	Polyclonal	1:1000
α-SMA	Cell Signaling Technology, USA	19245T	D4K9N	1:1000
PDGF-AA	Abcam, USA	Ab216619	Polyclonal	1:1000
CIDEC	Novus Biologicals, USA	NB100-430SS	Polyclonal	1:1000
β-ACTIN	ZSGB-BIO, China	TA-09	OTI1	1:2000
CIDEA	Proteintech, USA	13170-1-AP	Polyclonal	1:1000
COL1A1	Abcam, USA	Ab34710	Polyclonal	1:1000
p-AKT (S473)	Bioworld Technology, USA	BS4007	Polyclonal	1:1000
p-AKT (T308)	Bioworld Technology, USA	AP0056	Polyclonal	1:1000
AKT	Bioworld Technology, USA	AP0059	Polyclonal	1:1000

p-RhoA (S188)	Abcam, USA	Ab41435	Polyclonal	1:1000
RhoA	Abcam, USA	Ab187027	EPR18134	1:1000
F4/80	Cell Signaling Technology, USA	70076T	D2S9R	1:1000
ATF3	Abcam, USA	Ab207434	EPR19488	1:1000
COL3A1	Proteintech	22734-1-AP	Polyclonal	1:1000
RhoA	Cell Signaling Technology, USA	2117T	67B9	1:1000
RhoB	Cell Signaling Technology, USA	2098T	Polyclonal	1:1000
RhoC	Cell Signaling Technology, USA	3430T	D40E4	1:1000
RAC1/2/3	Cell Signaling Technology, USA	2465T	Polyclonal	1:1000
p-RAC1	Cell Signaling Technology, USA	2461T	Polyclonal	1:1000
CDC42	Cell Signaling Technology, USA	2466T	11A11	1:1000
PI3 Kinase p85	Cell Signaling Technology, USA	4257	19H8	1:1000
Rabbit Anti-	Abcam	Ab6728	Polyclonal	1:5000

Mouse IgG H&L				
Goat Anti-Rabbit IgG H&L	Abcam	Ab6721	Polyclonal	1:5000

Supplementary References

1. Tuo, L. *et al.* PCK1 negatively regulates cell cycle progression and hepatoma cell proliferation via the AMPK/p27Kip1 axis. *J. Exp. Clin. Cancer Res.* **38**, 50 (2019).
