# Supplementary material for: Deficiency of gluconeogenic enzyme PCK1 promotes metabolic-associated fatty liver disease through PI3K/AKT/PDGF axis activation in male mice
Source: Nat Commun. 2023 Mar 14;14:1402. doi: 10.1038/s41467-023-37142-3 (PMC10015095; doi:10.1038/s41467-023-37142-3)
Supplement: Supplementary file 3 — Reporting Summary [file 41467_2023_37142_MOESM3_ESM.pdf]

nature portfolio

Corresponding author(s): Kai Wang. Ailons Huang and Ni Tang

Last updated by author(s): Feb 22, 2023

Reporting Summary

Nature Portfolio wishes to improve the reproducibility of the work that we publish. This form provides structure for consistency and transparency in reporting. For further information on Nature Portfolio policies, see our Editorial Policies and the Editorial Policy Checklist.

Statistics

For all statistical analyses, confirm that the following items are present in the figure legend, table legend, main text, or Methods section.

n/a	Confirmed	
☐	☒	The exact sample size (*n*) for each experimental group/condition, given as a discrete number and unit of measurement
☐	☒	A statement on whether measurements were taken from distinct samples or whether the same sample was measured repeatedly
☐	☒	The statistical test(s) used AND whether they are one- or two-sided<br>*Only common tests should be described solely by name; describe more complex techniques in the Methods section.*
☒	☐	A description of all covariates tested
☐	☒	A description of any assumptions or corrections, such as tests of normality and adjustment for multiple comparisons
☐	☒	A full description of the statistical parameters including central tendency (e.g. means) or other basic estimates (e.g. regression coefficient) AND variation (e.g. standard deviation) or associated estimates of uncertainty (e.g. confidence intervals)
☐	☒	For null hypothesis testing, the test statistic (e.g. *F*, *t*, *r*) with confidence intervals, effect sizes, degrees of freedom and *P* value noted<br>*Give P values as exact values whenever suitable.*
☒	☐	For Bayesian analysis, information on the choice of priors and Markov chain Monte Carlo settings
☒	☐	For hierarchical and complex designs, identification of the appropriate level for tests and full reporting of outcomes
☒	☐	Estimates of effect sizes (e.g. Cohen's *d*, Pearson's *r*), indicating how they were calculated

Our web collection on statistics for biologists contains articles on many of the points above.

Software and code

Policy information about availability of computer code

Data collection	Confocal microscopy: Leica TCS SP8 Laser Scanning Confocal Microscope; RT-PCR: CFX Connect Real-time System (BIO-RAD)
Data analysis	R 3.6.3 software; GraphPad Prism 8.3.0; Image J version 2×.2.1.4.7

For manuscripts utilizing custom algorithms or software that are central to the research but not yet described in published literature, software must be made available to editors and reviewers. We strongly encourage code deposition in a community repository (e.g. GitHub). See the Nature Portfolio guidelines for submitting code & software for further information.

Data

Policy information about availability of data

All manuscripts must include a data availability statement. This statement should provide the following information, where applicable:

- Accession codes, unique identifiers, or web links for publicly available datasets
- A description of any restrictions on data availability
- For clinical datasets or third party data, please ensure that the statement adheres to our policy

RNA-seq data that support the findings of this study have been deposited in GEO under accession code GSE162211. (https://www.ncbi.nlm.nih.gov/geo/query/acc.cgi?acc=GSE162211).The previously published data sets re-analysed in this study were obtained from [Gene Expression Omnibus (GEO)], through the accession code [GSE126848, GSE89632, and GSE135251] [GEO Accession viewer (nih.gov); GEO Accession viewer (nih.gov); GEO Accession viewer (nih.gov)]. The untargeted

metabolomic data reported in this article have been deposited in the OMIX, China National Center for Bioinformation / Beijing Institute of Genomics, Chinese Academy of Sciences (https://ngdc.cncb.ac.cn/omix: accession no. OMIX003062) OMIX (cncb.ac.cn) .

Human research participants

Policy information about studies involving human research participants and Sex and Gender in Research.

Reporting on sex and gender

Total 46 patients, including 23 women and 23 men, participated in one studies: human NASH characterization.

Population characteristics

The clinical information and histologic features of participants included in this study was shown in Supplementary Table 1. The information was also shown below.

	Control	NASH
N (male/female)	10(5/5)	36(18/18)
Age (years)	43.7 ± 4.94	44.64 ± 2.81
BMI (kg/m2)	21.34 ± 0.92	27.54 ± 0.76
ALT (U/L)	21.5 ± 4.54	136.2 ± 11.47
AST (U/L)	23.4 ± 1.56	74 ± 6.52
γ-GT (U/L)	18.6 ± 1.69	83.17 ± 10.49
TC (mmol/L)	2.30 ± 0.20	4.56 ± 0.20
TG (mmol/L)	1.09 ± 0.15	1.41 ± 0.12
Glucose (mmol/L)	4.71 ± 0.17	7.79 ± 0.38

Recruitment

All the participants in this study were enrolled at Chongqing Medical University and Xin Hua Hospital. No expect biases, including self-selection bias was expected from patient samples. The investigation conforms to the principles that are outlined in the Declaration of Helsinki regarding the use of human tissues.

Ethics oversight

The present study were approved by the Research Ethics Committee of the Institutional Ethics Committees of Chongqing Medical University and Xin Hua Hospital

Note that full information on the approval of the study protocol must also be provided in the manuscript.

Field-specific reporting

Please select the one below that is the best fit for your research. If you are not sure, read the appropriate sections before making your selection.

☒ Life sciences ☐ Behavioural & social sciences ☐ Ecological, evolutionary & environmental sciences

For a reference copy of the document with all sections, see nature.com/documents/nr-reporting-summary-flat.pdf

Life sciences study design

All studies must disclose on these points even when the disclosure is negative.

Sample size

The sample sizes for this study were chosen based on generally expected variations of metabolic parameters and typical sample sizes for metabolic studies documented in literature (References: PMID: 28437865; 30855276; 30855276; 31857195; 29705240, 30613274 and 34893641). That allows to determine statistical differences in animal experiments and in vitro cell culture experiments. Sample numbers were well described in the Figure legends.

Data exclusions

No data were excluded from the study

Replication

All experimental findings were reproduced as stated in figure legends. All additional replication attempts were successful.

Randomization

All samples were randomly allocated

Blinding

The experiments were not blinded. However, we followed standard laboratory procedures of randomization. Each experiment was associated with the proper controls, and compared samples were collected and analyzed under the same condition.

Reporting for specific materials, systems and methods

We require information from authors about some types of materials, experimental systems and methods used in many studies. Here, indicate whether each material, system or method listed is relevant to your study. If you are not sure if a list item applies to your research, read the appropriate section before selecting a response.

Materials & experimental systems

n/a	Involved in the study
☐	☒ Antibodies
☐	☒ Eukaryotic cell lines
☒	☐ Palaeontology and archaeology
☐	☒ Animals and other organisms
☒	☐ Clinical data
☒	☐ Dual use research of concern

Methods

n/a	Involved in the study
☒	☐ ChIP-seq
☒	☐ Flow cytometry
☒	☐ MRI-based neuroimaging

Antibodies

Antibodies used

Rabbit polyclonal anti-PCK1 (Bioworld Technology, cat#BS6870 1:2000 for immunoblotting and 1: 600 for immunohistochemistry)

Rabbit polyclonal anti-PPARa (Proteintech, cat#15540-1-AP 1:1000 for immunoblotting)

Rabbit monoclonal anti-CD36 (Abcam, cat#Ab133625;EPR6573; 1:1000 for immunoblotting)

Rabbit polyclonal anti-FATP1 (Affinity Biosciences, cat#DF7716 1:1000 for immunoblotting)

Rabbit monoclonal anti-a-SMA (Cell Signaling Technology, cat#19245T;D4K9N; 1:1000 for immunoblotting ,1: 400 for immunohistochemistry and 1:200 for immunofluorescence)

Rabbit polyclonal anti-PDGF-AA (Abcam, cat#Ab216619 1:1000 for immunoblotting and 1: 200 for immunohistochemistry)

Rabbit polyclonal anti-CIDEC (Novus Biologicals, cat#NB100-430SS 1:1000 for immunoblotting)

Mouse monoclonal anti-β-ACTIN (ZSGB-BIO, cat#TA-09;OTI1; 1:2000 for immunoblotting)

Rabbit polyclonal anti-CIDEA (Proteintech, cat#13170-1-AP 1:1000 for immunoblotting)

Rabbit polyclonal anti-COL1A1 (Abcam, cat# Ab34710 1:1000 for immunoblotting)

Rabbit polyclonal anti-p-AKT (S473) (Bioworld Technology, cat#BS4007 1:1000 for immunoblotting and 1: 200 for immunohistochemistry)

Rabbit polyclonal anti-p-AKT (T308) (Bioworld Technology, cat#AP0056 1:1000 for immunoblotting and 1: 200 for immunohistochemistry)

Rabbit polyclonal anti-AKT (Bioworld Technology, cat#APO059 1:1000 for immunoblotting)

Rabbit polyclonal anti-p-RhoA (S188) (Abcam, cat#Ab41435 1:1000 for immunoblotting and 1: 200 for immunohistochemistry)

Rabbit monoclonal anti-RhoA (Abcam, cat#Ab187027;EPR18134; 1:1000 for immunoblotting)

Rabbit monoclonal anti-RhoA (Cell Signaling Technology, USA, 2117T;E67B9; 1:1000 for immunoblotting)

Rabbit monoclonal anti-F4/80 (Cell Signaling Technology, cat#70076T;D2S9R; 1:1000 for immunoblotting and 1: 200 for immunohistochemistry)

Rabbit monoclonal anti-ATF3 (Abcam, cat#Ab207434;EPR19488; 1:1000 for immunoblotting and 1: 200 for immunohistochemistry)

Rabbit polyclonal anti-COL3A1 (Proteintech cat#22734-1-AP 1:1000 for immunoblotting and 1: 200 for immunohistochemistry)

Rabbit polyclonal anti-RhoB (Cell Signaling Technology, cat#2098T 1:1000 for immunoblotting)

Rabbit polyclonal anti-RhoC (Cell Signaling Technology, cat#3430T;D40E4; 1:1000 for immunoblotting)

Rabbit polyclonal anti-RAC1/2/3 Cell Signaling Technology, cat#2465T 1:1000 for immunoblotting)

Rabbit polyclonal anti-p-RAC1 (Cell Signaling Technology, cat#2461T 1:1000 for immunoblotting)

Rabbit polyclonal anti-CDC42 (Cell Signaling Technology, cat#2466T;11A11; 1:1000 for immunoblotting)

Rabbit monoclonal anti-PI3 Kinase p85 (Cell Signaling Technology, cat#4257;19H8; 1:1000 for immunoblotting and 1: 50 for immunoprecipitation)

Rabbit polyclonal Anti-Mouse IgG H&L (Abcam, Ab6728 1:5000 for immunoblotting)

Goat polyclonal Anti-Rabbit IgG H&L (Abcam, Ab6721 1:5000 for immunoblotting)

Validation

All antibodies sourced from commercial corporation are well validated by the manufacturer and are widely used in the scientific community for Western Blotting and immunohistochemistry.

Rabbit polyclonal anti-PCK1 Bioworld Technology, cat#BS6870
Validated in our papers published before.
https://www.bioworlde.com/Primary-Antibodies/54987.html
Applications:Immunohistochemistry (IHC), Western Blotting (WB)
Reactivity:Homo sapiens (Human), Mus musculus (House mouse), Rattus norvegicus (Rat)

Rabbit polyclonal anti-PPARa Proteintech, cat#15540-1-AP
https://www.ptgcn.com/products/ PPARA-Antibody-15540-1-AP.htm
Validated by other users, cited 127 times in the company website
Applications: Enzyme-linked immunosorbent assay (ELISA), Immunohistochemistry (IHC), Immunoprecipitation (IP), Western Blotting (WB)
Reactivity: Homo sapiens (Human), Mus musculus (House mouse), Rattus norvegicus (Rat)

Rabbit monodonal anti-CD36 Abeam, cat#Ab133625
https://www.abcam.cn/ed36-antibody-l epr5573-ab133625.html
Validated by other users, cited 89 times in the company website
Applications: Immunohistochemistry-paraffin (IHC-P), Immunoprecipitation (IP), Western Blotting (WB)
Reactivity: Cavia porcellus (Guinea Pig), Homo sapiens (Human), Mus musculus (House mouse)

Rabbit polyclonal anti-FATP1 Affinity Biosciences, catDF771s
http://www.affbiotech.crl goods-11452-DF7716-FATP1 Antibody.htmi
Validated by other users, cited 2 times in the company website
Applications:Western Blotting (WB)
Reactivity:Homo sapiens (Human), Mus musculus (House mouse), Rattus norvegicus (Rat)

Rabbit monodonal anti-a-SMA Cell Signaling Technology, cat#19245T
https://www.cellsignal.cn/products/primary-antibodies/a-smooth-l muscle-actin-d4k9n-xp-rabbit-mab/192457site-searchtype=Products&N=4294956287&Ntt=19245t&fromPage=plp&_requestid=1063676.
Validated by other users, cited 279 times in the company website
Applications: Immunohistochemistry-paraffin (IHC-P) Immunoprecipitation (IP)
Reactivity: H- Human M- Mouse R- Rat Hm- Hamster Mk- Monkey Vir- Virus Mi- Mink C- Chicken Dm- Drosophila melanogaster X-Xenopus Z- Zebrafish B- Bovine DG- Dog PG- Pig Sc- Saccharomyces cerevisiae Ce- Caenorhabditis elegans Hr- Horse GP- Guinea pig Rab-Rabbit All- Expected for all species.

Rabbit polyclonal anti-PDGF-AA Abcam, cat#Ab216619
https://www.abcam.cn/pdgf-aa-antibody-ab216619.html
Validated by other users, cited 3 times in the company website
Applications:Immunohistochemistry-paraffin (IHC-P), Western Blotting (WB)
Reactivity:Homo sapiens (Human), Mus musculus (House mouse), Rattus norvegicus (Rat)

Rabbit polyclonal anti-CIDEC Novus Biologicals, cat#NB100-430ss
https://www.novusbio.com/l products/cidet-antibody nb100-430ss
Validated by other users, cited 14 times in the company website
Applications: Immunocytochemistry-immunofluorescence (ICC-IF), Western Blotting (WB)
Reactivity: None Available

Mouse monoclonal anti-beta-ACTIN ZSGB-BIO, cat#TA-09
Validated in our papers published before
Validated by other users, cited 7 times in the company website
Applications: WB
Reactivity:Human, mouse, rat, monkey, dog (Predicted: pig, chicken, bovine, rabbit, chimpanzee, horse, xenopus)

Rabbit polyclonal anti-CIDEA Proteintech, cat#13170-1-AP
https://www.ptglab.com/products/CIDEA-Antibody-13170-1-AP.htm
Applications: Enzyme-linked immunosorbent assay (ELISA), Western Blotting (WB)
Reactivity:Homo sapiens (Human), Mus musculus (House mouse), Rattus norvegicus (Rat)

Rabbit polyclonal anti-COL1A1 Abcam, cat# Ab34710
https://www.abcam.cn/collagen-i-antibodyab34710 html Validated by other users, cited 1342 times in the company website
Applications: Enzyme-linked immunosorbent assay (ELISA), Immunocytochemistry-immunofluorescence (ICC-IF), Immunohistochemistry (IHC), Immunohistochemistry-paraffin (IHC-P), Immunoprecipitation (IP), Western Blotting (WB)
Reactivity: Bos taurus (Bovine), Callithrix jacchus (Common marmoset), Capra aegagrus hircus (Goat), Equus caballus (Horse), Homo sapiens (Human), Mus musculus (House mouse), Ovis aries (Sheep), Rattus norvegicus (Rat), Sus scrofa domesticus (Pig)

Rabbit polyclonal anti-p-AKT (S473) Bioworld Technology, cat#BS4007
Validated in our papers published before.
Applications: Immunohistochemistry (IHC), Western Blotting (WB)
Reactivity: Homo sapiens (Human), Mus musculus (House mouse), Rattus norvegicus (Rat)

Rabbit polyclonal anti-p-AKT (T308) Bioworld Technology, cat#AP0056 1:1000 for immumoblotting) Validated in our papers published before. https://www.bioworlde.com/Primary-Antibodies/45319.html
Applications:Immunohistochemistry (IHC), Western Blotting (WB)

Reactivity:Homo sapiens (Human),Mus musculus (House mouse),Rattus norvegicus (Rat)

Rabbit polyclonal anti-AKT (Bioworld Technology, cat#AP0059 1:1000 for immunoblotting) Validated in our papers published before.
https://www.bioworlde.com/Primary-Antibodies/48550.html
Reactivity: Human,Mouse,Rat
Applications: WB,IF

Rabbit polyclonal anti-p-RhoA (5188) Abcam, cat#Ab41435
https://www.abcam.com/rhoa-phospho-s188-antibody-ab41435.html
Validated in our papers published before.
Applications:Enzyme-linked immunosorbent assay (ELISA), Western Blotting (WB)
Reactivity: Homo sapiens (Human), Mus musculus (House mouse), Rattus norvegicus (Rat)

Rabbit monodonal anti-RhoA (Abcam, cat#Ab187027 1:1000 for immunoblotting) https://www.abcam.cn/rhoa-antibody-lepr18134-ab187027.html Validated by other users, cited 62 times in the company website
Applications: Flow cytometry/Cell sorting (FC/FACS), Immunocytochemistry-immunofluorescence (ICC-IF), Immunohistochemistry-paraffin (IHC-P), Western Blotting (WB)
Reactivity: Homo sapiens (Human), Mus musculus (House mouse), Rattus norvegicus (Rat)

Rabbit monoclonal anti-RhoA Cell Signaling Technology, USA, 2117T;E67B9
Application: WB- Western, Blotting IP- Immunoprecipitation, IHC- Immunohistochemistry, ChIP- chromatin immunoprecipitation eCLIP-eCLIP, IF- Immunofluorescence, F-flow cytometry
Reactivity: H- Human, M- Mouse, R- Rat, Hm- Hamster, Mk- Monkey, Vir- Virus, Mi- Mink, C- Chicken, Dm- Drosophila, melanogaster X- Xenopus, Z- Zebrafish, B- Bovine, DG- Dog, PG- Pig, Sc- Saccharomyces cerevisiae, Ce- Caenorhabditis elegans, Hr- Horse, GP-Guinea pig, Rab-Rabbit, All- Expected for all species

Rabbit monodonal anti-F4/80 Cell Signaling Technology, cat#70076T
https://www.cellsignal.cn/products/primary-antibodies/f4-80-d2s9r-xp-rabbit-mab/70076?site-search-type=Products&N=4294956287&Ntt=70076t&fromPage=plp&_requestid=1372996
Validated by other users, cited 159 times in the company website
Application :WB- Western Blotting, IP- Immunoprecipitation, IHC- Immunohistochemistry, ChIP- chromatin immunoprecipitation, eCLIP-eCLIP, IF- Immunofluorescence, F-flow cytometry
Reactivity: H- Human, M- Mouse, R- Rat, Hm- Hamster, Mk- Monkey, Vir- Virus, Mi- Mink, C- Chicken, Dm- Drosophila melanogaster X- Xenopus, Z- Zebrafish, B- Bovine, DG- Dog, PG- Pig, Sc- Saccharomyces cerevisiae, Ce- Caenorhabditis elegans, Hr- Horse, GP-Guinea pig Rab-Rabbit, All- Expected for all species

Rabbit monodonal anti-ATF3 Abcam, cat#Ab207434
https://l www.abcam.cn/atf3-antibody-epr19488-chip-grade-ab207434.html Validated by other users, cited 19 times in the company website
Applications :Chromatin immunoprecipitation (ChIP), Immunocytochemistry-immunofluorescence (ICC-IF), Immunoprecipitation (IP), Western Blotting (WB)
Reactivity: Homo sapiens (Human), Mus musculus (House mouse)

Rabbit polycional anti-COL3A1 Proteintech cat#22734-1-AP
https://www.ptgon.com/products/COL3A1-Antibody-22734-1-AP.htm Validated by other users, cited 207 times in the company website
Applications: Enzyme-linked immunosorbent assay (ELISA), Immunohistochemistry (IHC), Immunoprecipitation (IP), Western Blotting (WB)
Reactivity: Homo sapiens (Human), Mus musculus (House mouse), Rattus norvegicus (Rat)

Rabbit polyclonal anti-RhoB Cell Signaling Technology, cat#2098T
https://www.celisignal.cn/l products/primary-antibodies/rhob-antibody/20987-16568495931778Ntt-2098t&tahead-true Validated by other users, cited 6 times in the company website
Application :WB- Western Blotting, IP- Immunoprecipitation, IHC- Immunohistochemistry, ChIP- chromatin immunoprecipitation, eCLIP-eCLIP, IF- Immunofluorescence, F-flow cytometry
Reactivity :H- Human, M- Mouse, R- Rat, Hm- Hamster, Mk- Monkey, Vir- Virus, Mi- Mink, C- Chicken, Dm- Drosophila melanogaster X- Xenopus, Z- Zebrafish ,B- Bovine, DG- Dog, PG- Pig, Sc- Saccharomyces cerevisiae Ce- Caenorhabditis elegans, Hr- Horse, GP-Guinea pig Rab-Rabbit, All- Expected for all species

Rabbit polyclonal anti-RhoC Cell Signaling Technalogy, cat#3430T
https://www.celisignal.on/l products/primary-antibodies/rhoc-d4De4-rabbit-mab/3430/site-searchtype-Products&N-4294956287&Ntt-3430t&fromPage-plp&_requestid1067608
Validated by other users, cited 59 times in the company website
Application:WB- Western Blotting, IP- Immunoprecipitation, IHC- Immunohistochemistry, ChIP- chromatin immunoprecipitation, eCLIP-eCLIP, IF- Immunofluorescence, F-flow cytometry
Reactivity :H- Human M- Mouse R- Rat Hm- Hamster Mk- Monkey Vir- Virus Mi- Mink C- Chicken Dm- Drosophila melanogaster X-Xenopus Z- Zebrafish B- Bovine DG- Dog PG- Pig Sc- Saccharomyces cerevisiae Ce- Caenorhabditis elegans Hr- Horse GP- Guinea pig Rab-Rabbit All- Expected for all species

Rabbit polyclonal anti-RAC1/2/3 Cell Signaling Technology, cat2465T
https://www.celisignal.cn/ products/primary-antibadies/rac1-2-3-antibody/24657site-searchtype-Products&N-4294956287&Ntt-2465t&fromPage-plp&_requestid-1068177
Validated by other users, cited 91 times in the company website
Application:WB- Western Blotting, IP- Immunoprecipitation, IHC- Immunohistochemistry, ChIP- chromatin immunoprecipitation eCLIP-eCLIP, IF- Immunofluorescence, F-flow cytometry
Reactivity :H- Human, M- Mouse, R- Rat, Hm- Hamster, Mk- Monkey, Vir- Virus, Mi- Mink, C- Chicken, Dm- Drosophila melanogaster

X- Xenopus, Z- Zebrafish, B- Bovine, DG- Dog, PG- Pig, Sc- Saccharomyces cerevisiae, Ce- Caenorhabditis elegans, Hr- Horse, GP- Guinea pig, Rab-Rabbit All- Expected for all species

Rabbit polyclonal anti-p-RAC1 Cell Signaling Technology, cat2461T
https://www.cellsignal.cn/ products/primary antibodies/phospho rac1 cdc42-ser71-antibody/24617site searchtype-Products&N-4294956287&Ntt-2461t&fromPage-plp&_requestid-1068463
Validated by other users, cited 40 times in the company website
Application :WB- Western, Blotting IP- Immunoprecipitation, IHC- Immunohistochemistry, ChIP- chromatin immunoprecipitation, eCLIP-eCLIP, IF- Immunofluorescence, F-flow cytometry
Reactivity : H- Human M- Mouse R- Rat Hm- Hamster Mk- Monkey Vir- Virus Mi- Mink C- Chicken Dm- Drosophila melanogaster X-Xenopus Z- Zebrafish B- Bovine DG- Dog PG- Pig Sc- Saccharomyces cerevisiae Ce- Caenorhabditis elegans Hr- Horse GP- Guinea pig Rab-Rabbit All- Expected for all species

Rabbit polyclonal anti-CDC42 Cell Signaling Technology, cat#2466T
https://www.cellsignal.cn/l products/primary-antibodies/cdc42-11a11-rabbit-mab/2466?site-searchtype=Products&N=4294956287&Ntt=2466t&fromPage=plp&_requestid=1068589
Validated by other users, cited 79 times in the company website
Application :WB- Western Blotting IP- Immunoprecipitation IHC- Immunohistochemistry ChIP- chromatin immunoprecipitation C&R - CUT&RUN C&T-Cut & TAG DB - Dot blot eCLIP-eCLIP IF- Immunofluorescence F-flow cytometry
Reactivity: H- Human M- Mouse R- Rat Hm- Hamster Mk- Monkey Vir- Virus Mi- Mink C- Chicken Dm- Drosophila melanogaster X-Xenopus Z- Zebrafish B- Bovine DG- Dog PG- Pig Sc- Saccharomyces cerevisiae Ce- Caenorhabditis elegans Hr- Horse GP- Guinea pig Rab-Rabbit All- Expected for all species

Rabbit monodional anti-PI3 Kinase p85 Cell Signaling Technology, cat#4257
https://www.cellsignal.cn/products/primary-antibodies/pi3-kinase-p85-19h8-rabbit-mab/4257?=1656850681699&Ntt=4257&tahead=true
Validated by other users, cited 405 times in the company website
Application :WB- Western Blotting, IP- Immunoprecipitation, IHC- Immunohistochemistry, ChIP- chromatin immunoprecipitation eCLIP-eCLIP, IF- Immunofluorescence, F-flow cytometry
Reactivity : H- Human, M- Mouse, R- Rat, Hm- Hamster, Mk- Monkey, Vir- Virus, Mi- Mink C- Chicken Dm- Drosophila, melanogaster X- Xenopus, Z- Zebrafish, B- Bovine, DG- Dog, PG- Pig, Sc- Saccharomyces cerevisiae Ce- Caenorhabditis elegans, Hr- Horse, GP-Guinea pig, Rab-Rabbit, All- Expected for all species

Rabbit Anti-Mouse IgG H&L Abcam, Ab6728
https://www.abcam.com/rabbit-mouse-igg-hl-hrp-ab6728.html
Applications: Dot blot Enzyme-linked immunosorbent assay (ELISA)Immunocytochemistry-immunofluorescence (ICC-IF)Immunohistochemistry (IHC)Immunohistochemistry-paraffin (IHC-P)Western Blotting (WB)

Goat Anti-Rabbit IgG H&L Abcam, Ab6721
Applications:Dot blotEnzyme-linked immunosorbent assay (ELISA)Immunocytochemistry (ICC)Immunohistochemistry (IHC)Immunohistochemistry-paraffin (IHC-P)Western Blotting (WB)
https://www.abcam.com/goat-rabbit-igg-hl-hrp-ab6721.html

Eukaryotic cell lines

Policy information about cell lines and Sex and Gender in Research

Cell line source(s)	the human hepatic stellate cell (HSC) line LX-2 and human hepatocyte line MIHA cells (provided by Dr Ben C.B. Ko, The Hong Kong Polytechnic University, Hongkong, China); HEK-293T was provided by Prof. Bing Sun (Center for Excellence in Molecular Cell Science, Chinese Academy of Sciences, China).
Authentication	The cell lines have not been authenticated recently.
Mycoplasma contamination	The cell lines were tested negative for mycoplasma contamination.
Commonly misidentified lines (See ICLAC register)	No commercially misidentified cells were used.

Animals and other research organisms

Policy information about studies involving animals; ARRIVE guidelines recommended for reporting animal research, and Sex and Gender in Research

Laboratory animals	Male mice were used for experiments. Age is between 7 to 9 weeks. The age of mice for each experiment was shown in the relevant figure legends. Both Pck1f/f mice was on a mixed C57BL/6; 129S6/SvEv background. Alb-Cre and cPtenf/fPck1f/f mice were in C57BL/6J background. All mice were housed in temperature-controlled (23 °C) pathogen-free facilities with a 12 h light-dark cycle and humidity (50% ± 10%) conditions.
Wild animals	This study did not involve wild animals.
Reporting on sex	Male mice were used in this study. Use of male mice have been discussed in the Discussion section in this study.
Field-collected samples	This study did not involve samples collected from the field.

Ethics oversight | Animal experiments were approved by the Animal Experimentation Ethics Committees of Chongqing Medical University

Note that full information on the approval of the study protocol must also be provided in the manuscript.
